# Research Progress on Chemical Constituents of *Zingiber officinale* Roscoe

**DOI:** 10.1155/2019/5370823

**Published:** 2019-12-20

**Authors:** Yan Liu, Jincheng Liu, Yongqing Zhang

**Affiliations:** ^1^School of Pharmacy, Shandong University of Traditional Chinese Medicine, Jinan 250355, China; ^2^School of Chemical and Chemical Engineering, Shandong University of Technology, Zibo 255049, China

## Abstract

*Zingiber officinale* Roscoe is commonly used in food and pharmaceutical products but can also be used in cosmetics and daily necessities. In recent years, many scholars have studied the chemical composition of *Zingiber officinale* Roscoe; therefore, it is necessary to comprehensively summarize the chemical composition of *Zingiber officinale* Roscoe in one article. The purpose of this paper is to provide a comprehensive review of the chemical constituents of *Zingiber officinale* Roscoe. The results show that *Zingiber officinale* Roscoe contains 194 types of volatile oils, 85 types of gingerol, and 28 types of diarylheptanoid compounds, which can lay a foundation for further applications of *Zingiber officinale* Roscoe.

## 1. Introduction

*Zingiber officinale* Roscoe (ZOR, also Shengjiang in Chinese) is a perennial herb from the Zingiberaceae family, native to the Pacific Islands. It can be found in the Chinese provinces of Shandong, Henan, Hubei, Yunnan, Guangdong, Sichuan, and Jiangsu. ZOR is the fresh root of ginger, which is not only an important condiment but also one of the most commonly used Chinese medicines in clinical practice. Traditional Chinese medicine believes that ZOR has effects of releasing exterior and dissipating cold, arresting vomiting, resolving phlegm, and relieving coughs and can be used to treat fish and crab poison, stomach colds and vomiting, and cold sputum cough [1]. Modern pharmacological studies have shown that ZOR can promote digestion, improve blood circulation, lower blood lipids, lower blood sugar, relieve vestibular stimulation, and provide anti-inflammatory, antitumor, antimicrobial, and antioxidant effects [2–5]. Due to its rich active constituents, ZOR has been used in cosmetics [6], toothpaste [7], and health foods [8–10].

All development and utilization of ZOR are based on its material composition. The chemical composition of ZOR is complex, includes more than 300 types of species, and can be broadly divided into three categories: volatile oils, gingerol, and diarylheptanoids [11–13]. In this paper, the existing research literature of ZOR is systematically summarized, and each chemical composition and its chemical structure are listed in detail, with a view to providing references for quality control, cultivation production, and further development of ZOR.

## 2. Constituents

### 2.1. Volatile Oils

Volatile oils, also known as ginger essential oils, are generally composed of terpenoids [14]. Ginger essential oils give ZOR a unique aromatic smell [11]. The volatile oil composition varies based on where the ZOR is harvested. Currently, the ingredients identified in the volatile oils of ZOR and their chemical structures are shown in Table 1.

### 2.2. Gingerol

Gingerol is the spicy component of ZOR. It is a mixture of various substances, all of which contain the 3-methoxy-4-hydroxyphenyl functional group. Gingerols can be divided into gingerols, shogaols, paradols, zingerones, gingerdiones, and gingerdiols, according to the different fatty chains connected by this functional group [28, 29]. The structural formulas are given in Table 2.

### 2.3. Diarylheptanoids

Diarylheptanoid is a group of compounds with 1,7-disubstituted phenyl groups and heptane skeletons in its parent structure. Currently, it can be divided into linear diphenyl heptane and cyclic diphenyl heptane compounds with antioxidant activity [53]. The structural formulas are shown in Table 3.

### 2.4. Others

#### 2.4.1. Proteins and Amino Acids

ZOR contains a variety of amino acids, including glutamate, aspartic acid, serine, glycine, threonine, alanine, cystine, valine, methionine, isoleucine, leucine, tyrosine, phenylalanine, lysine, histidine, arginine, proline [22, 60], and tryptophan [51].

#### 2.4.2. Sugars

ZOR also contains polysaccharides [44], cellulose, and soluble sugar.

#### 2.4.3. Organic Acids

ZOR contains oxalic acid, tartaric acid, lactic acid, acetic acid, citric acid, succinic acid, formic acid, and malonic acid [61].

#### 2.4.4. Inorganic Elements

ZOR has been shown to contain more than 20 inorganic elements such as K, Mg, Ga, Mn, P, Al, Zn, Fe, and Ba [44].

## 3. Discussion

Various gingers have different regions and chemical compositions. Jolad [30] conducted quantitative analysis on the extracts of dichloromethane from Chinese white ginger and Japanese turmeric and found that the highest content of 6-gingerol was 28% and 34%, respectively. The next highest concentrations were 8-gingerol and 10-gingerol, and the lowest content of 6-shogaol was only 0.35%. Onyenekwe [62] determined that the main components of the volatile oils of Nigerian ginger were terpenoids such as zingiberene (29.5%) and *β-*sesquiphellandrene (18.4%), which were quite different from those of ginger grown in other regions. Another study showed the volatile oil content of ginger grown in five different areas of China (Shandong Laiwu, Anhui Tongling, Shandong Anqiu, Guangdong Guangzhou, and Hunan Rucheng) was 0.13%, 0.23%, 0.30%, 0.14%, and 0.17% [63], respectively. 6-Gingerol is often the quality standard for ginger, where the ginger found in Qianwei, Sichuan Province, shows a higher effective content of 6-gingerol than that of the pharmacopoeia standard of the People's Republic of China [64, 65]. The concentrations of 6-gingerol and 6-zingiberol of ginger grown in different regions of China vary greatly, which may be related to the growth environment [66]. Mature and fresh ginger extracts contain the same chemical components, but the difference is in the relative content of each component. Ginger oleoresin in mature ginger is significantly higher than that in fresh ginger. In aromatic terpenoids, the contents of 2-acetoxy-1,8-cineole, *β-*citronellal, citral, geraniol, geranyl acetate, and zingiberene in mature ginger are lower than those in fresh ginger. The relative content of *α*-curcumene in mature ginger was higher than that in fresh ginger. In spicy gingerol compounds, the relative content of gingerol in mature ginger is higher than that in fresh ginger, which may be the result of further synthesis and accumulation of gingerol components in the process of continued growth of mature ginger in the second year [48]. The varieties of ginger with the highest oil content are Laiwu ginger, Japanese ginger, Shannong 1 ginger, Shannong 2 ginger, and Anqiu big ginger, with concentrations of 4.56%, 4.42%, 4.52%, 4.50%, and 4.35%, respectively. Average oil contents of 3.45% and 3.16% were found in Jinchang ginger and Chinger, respectively. The lowest oil extraction rates were found in Anqiu small ginger, Fangzhou ginger, and Jinshi ginger, which were 2.95%, 2.60%, and 1.55%, respectively [48].

Ginger, as a kind of food and medicine, has many functions, such as antioxidant, anti-inflammatory, antimicrobial, anticancer, antiobesity, antidiabetic, antinausea, antiemetic, antiallergic, neuroprotective, hepatoprotective, cardiovascular protective, and respiratory protective activities [67]. Currently, most studies of the bioactive components of ginger focus on ginger volatile oil, gingerol, shogaol, and zingerone compounds. Ginger essential oil can effectively improve the antioxidant capacity of the liver, reduce inflammatory response, and protect against fatty liver [68]. The antioxidant compounds in ginger are primarily gingerol and diarylheptanoid. Substituents on alkyl chains contribute to free radical scavenging and oxidation inhibition of lipids [69]. Antioxidant activity is typically derived from gingerols, shogaols, and some related phenolic ketone derivatives [70]. Gingerols are spicy ingredients in which 6-gingerol shows the highest biological activity, so 6-gingerol is often used as an indicator of ginger quality [71]. 6-Gingerol has been used to inhibit angiogenesis in vivo and in vitro [72]. It has been shown to have anticancer and antigastric ulcer properties while suppressing central nervous stimulation and various pharmacological activities [73, 74]. 6-Gingerol has been used to treat tumors by regulating the apoptosis gene by reversing the abnormal expression of tumor cell genes. It can also affect the apoptosis signal transduction pathway and induce apoptosis [75]. 8-Gingerol and 10-gingerol have good inhibitory effects on the activity of various tumor cells, where the inhibitory effects are somewhat different. The two may affect the phosphorylation level of the MAPK pathway proteins ERK and P38, leading to G1 phase arrest of breast cancer cells, thus applying inhibitory effects on the proliferation of tumor cells [76]. The main components of strong heart are gingerol and 6-shogaol [77]. The effects of 6-gingerol and 6-shogaol on blood pressure have been shown to induce a hypotensive effect at low doses, while high doses have shown a three-phase reaction. Initially, blood pressure drops rapidly, then rises, and then provides a hypotensive effect at later stages [78]. Ginger polysaccharide has biological activities such as antitumor, hypoglycemic, lipid-lowering, immune regulation, antivirus, and antifatigue [79].

## 4. Conclusion

ZOR is a widely used drug and food in clinical and daily life and has been used in the prevention and treatment of the digestive, circulatory, respiratory, and central nervous system diseases and other diseases. In this paper, the chemical constituents found in ZOR in recent years are summarized, and the results show that more than 300 chemical constituents are identified from the extracts of ZOR, including 194 types of volatile oil, 85 types of gingerol, and 28 types of diarylheptanoids compounds. From this, it can be clearly observed that ZOR has a complex chemical composition. The interactions between the components provide the clinical effects; therefore, it is necessary to further study the chemical composition and pharmacological action of ginger, for further applications. Exploring the mechanism by which different components perform the same effects is a new way to develop drugs in the future; for example, 4-terpineol and beta-sitosterol can act on the two targets of the 5-hydroxytryptamine receptor 3A and the mu-type opioid receptor, respectively, and provide corresponding therapeutic effects on diarrhea and dysentery. This can provide ideas for the research and development of new drugs and lay a foundation for further applications of ZOR.

## Figures and Tables

**Table 1:** Volatile oils in ZOR.

No.	Type	Name	Structure	Reference
1	Terpene	*α*-Terpinene	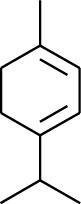	[15]
2	Terpene	*α*-Terpineol	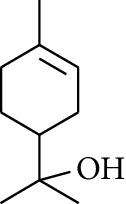	[15]
3	Terpene	4-Terpineol	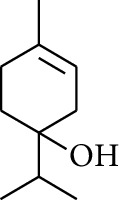	[15]
4	Terpene	Terpinolene	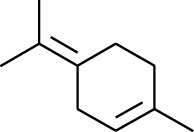	[15]
5	Terpene	*γ*-Terpinolene	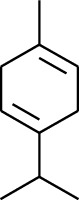	[15]
6	Alcohol	Cineole	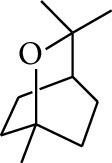	[15]
7	Alcohol	*β-*Eudesmol	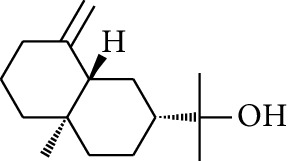	[15]
8	Alcohol	Nerol	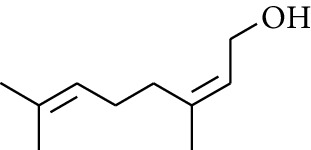	[15]
9	Alcohol	*trans-*Nerolidol	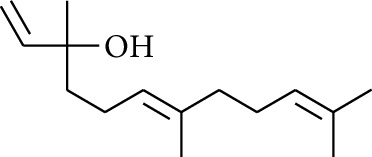	[15]
10	Alcohol	4-Isopropylbenzyl alcohol	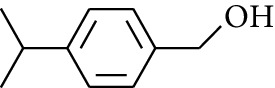	[15]
11	Alcohol	3,7-Dimethylocta-1,6-dien-3-ol	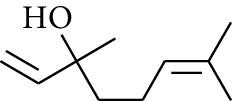	[15]
12	Alcohol	3,7-Dimethyloct-6-en-1-yn-3-ol	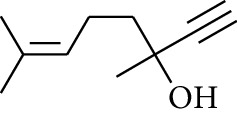	[15]
13	Alcohol	3-Methylhexan-2-ol	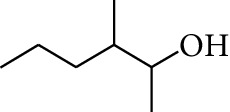	[15]
14	Alcohol	*cis-*Piperitol	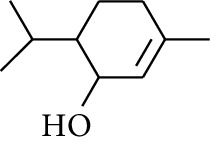	[15]
15	Alcohol	Borneol	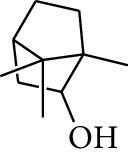	[15]
16	Alcohol	Elemol	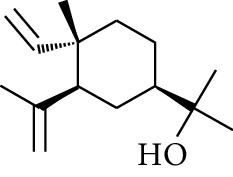	[15]
17	Alcohol	*tau-*Muurolol	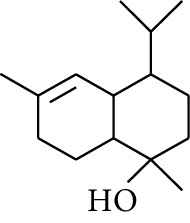	[15]
18	Alcohol	2-Methoxy-1,7,7-trimethylbicyclo[2.2.1]heptane	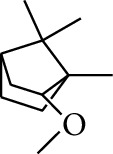	[15]
19	Alcohol	1-Isopropyl-4-methylcyclohex-3-enol	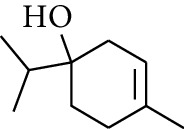	[15]
20	Alcohol	2-Tetradecanol	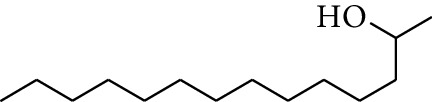	[15]
21	Alcohol	Myrtenol	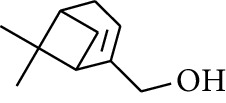	[15]
22	Alcohol	Citronellol	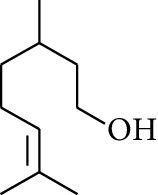	[15]
23	Alcohol	Geraniol	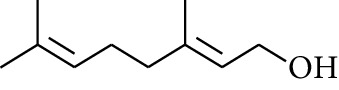	[15]
24	Alcohol	*cis-*Linalool oxide	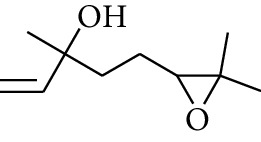	[15]
25	Alcohol	4-Ethoxybutan-1-ol	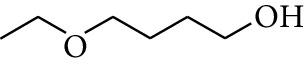	[15]
26	Alcohol	*α-*Eudesmol	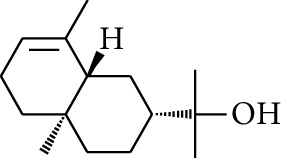	[15]
27	Alcohol	Nerolidol	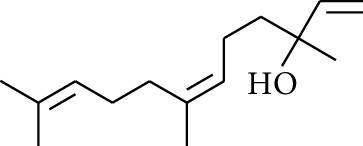	[15]
28	Alcohol	Farnesol	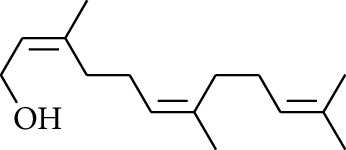	[15]
29	Alcohol	*trans-*4-Isopropyl-1-methyl-2-cyclohexen-1-ol	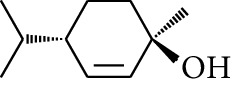	[15]
30	Alcohol	*cis-*4-Isopropyl-1-methyl-2-cyclohexen-1-ol	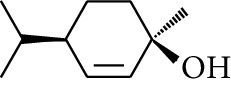	[15]
31	Alcohol	2-Heptanol	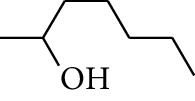	[16]
32	Alcohol	1-Methoxy-2-methyl	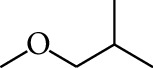	[16]
33	Alcohol	*cis-*Sesquisabinene hydrate	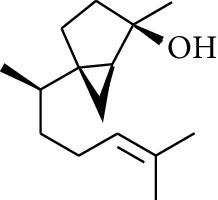	[17]
34	Alcohol	*cis-*2-*p*-Menthen-1-ol	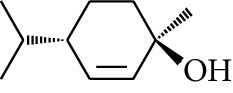	[17]
35	Alcohol	endo-Borneol	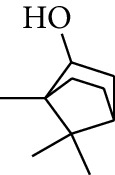	[17]
36	Alcohol	*trans*-Sabinene hydrate	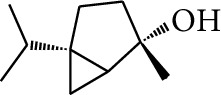	[17]
37	Alcohol	2-Nonanol	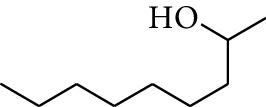	[18]
38	Alcohol	Propanol	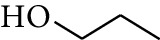	[18]
39	Alcohol	*cis-β-*Sesquiphellandrol	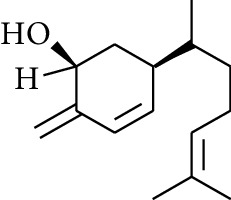	[18]
40	Alcohol	*trans-β*-Sesquiphellandrol	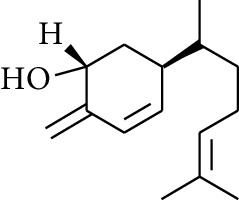	[18]
41	Alcohol	*β-*Santalol	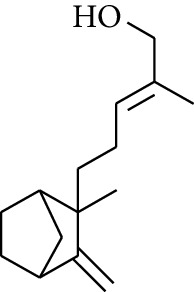	[19]
42	Alcohol	Zingiberol	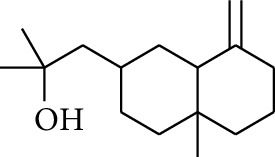	[19]
43	Alcohol	*tau-*Cadinol	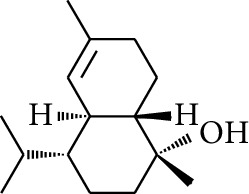	[20]
44	Alcohol	Zingiberenol	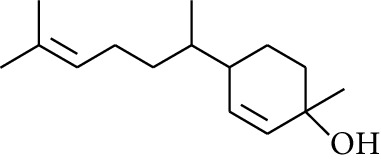	[21]
45	Alcohol	2-Pinen-5-ol	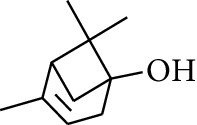	[21]
46	Alcohol	Bornyl methyl ether	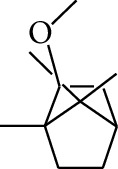	[21]
47	Alcohol	Isoborneol	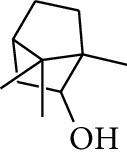	[22]
48	Alcohol	2-Decanol	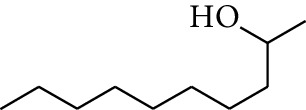	[22]
49	Alcohol	Fenchol	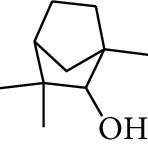	[22]
50	Alcohol	Linalool	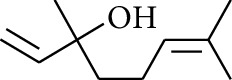	[23]
51	Alcohol	Plinol	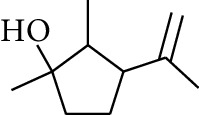	[23]
52	Alcohol	Camphenol	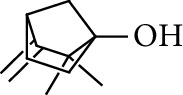	[23]
53	Alcohol	*trans-*2-Decen-1-ol	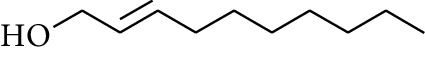	[23]
54	Alcohol	Hentriacontanol	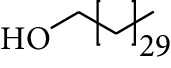	[24]
55	Alcohol	10-O-*β*-D-Glucopyranosyl-hydroxyl cineole	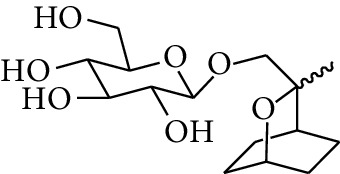	[25]
56	Aldoketone	Butanal	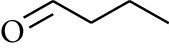	[15]
57	Aldoketone	Germacrone	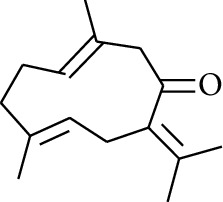	[15]
58	Aldoketone	2,6-Dimethylhept-5-enal	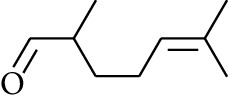	[15]
59	Aldoketone	2-Heptanone	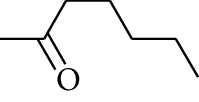	[15]
60	Aldoketone	(*E*)-Citral	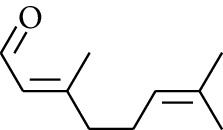	[15]
61	Aldoketone	(*Z*)-Citral	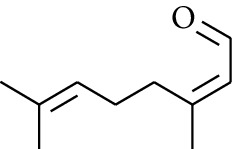	[15]
62	Aldoketone	2-Nonanone	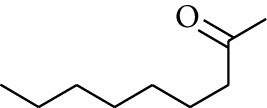	[15]
63	Aldoketone	3-((*3E*,*5E*)-Deca-3,5-dienyl)cyclopentanone	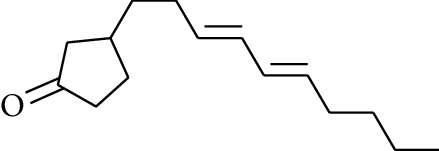	[15]
64	Aldoketone	*β-*Cyclocitral	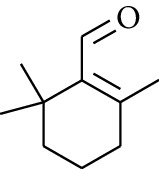	[15]
65	Aldoketone	2-Undecanone	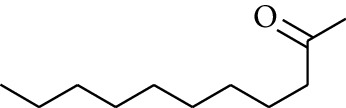	[15]
66	Aldoketone	1,7,7-Trimethylbicyclo[2.2.1]heptan-2-one	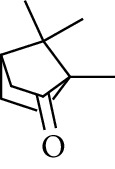	[15]
67	Aldoketone	*(1R)*-(*–*)-Myrtenal	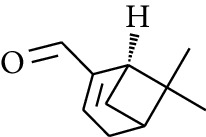	[15]
68	Aldoketone	*β-*Citronellal	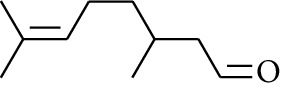	[15]
69	Aldoketone	Crypton	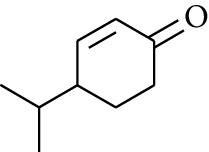	[15]
70	Aldoketone	4-Isopropylcyclohex-2-enone	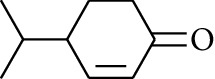	[15]
71	Aldoketone	Camphor	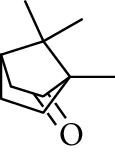	[15]
72	Aldoketone	6-Methyl-5-hepten-2-one	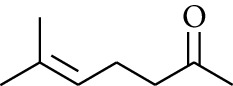	[15]
73	Aldoketone	*trans*,*trans-*Farnesal	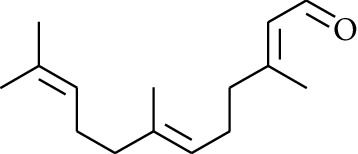	[15]
74	Aldoketone	Hexanal	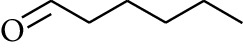	[16]
75	Aldoketone	Neral	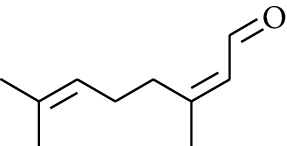	[17]
76	Aldoketone	Geranial	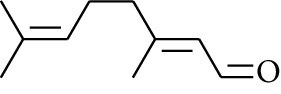	[17]
77	Aldoketone	Octanal	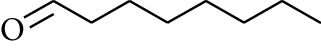	[17]
78	Aldoketone	Methyl heptenone	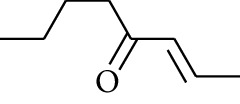	[18]
79	Aldoketone	Nonyl aldehyde	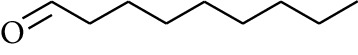	[18]
80	Aldoketone	Acetaldehyde	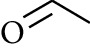	[18]
81	Aldoketone	Propionaldehyde	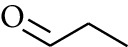	[18]
82	Aldoketone	Valeraldehyde	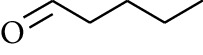	[18]
83	Aldoketone	Perillal	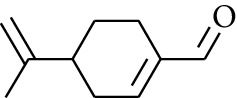	[19]
84	Aldoketone	(*E*)-Dodec-2-enal	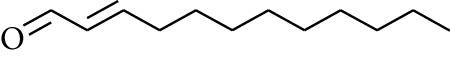	[21]
85	Aldoketone	(*Z*)-3,7-Dimethylocta-3,6-dienal	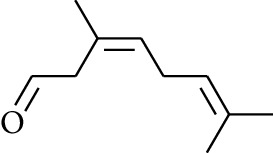	[21]
86	Aldoketone	(*E*)-3,7-Dimethylocta-3,6-dienal	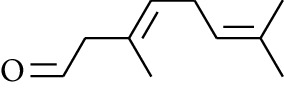	[21]
87	Aldoketone	(*E*)-Dec-2-enal	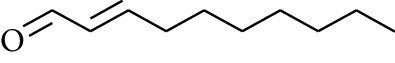	[21]
88	Aldoketone	Decanal	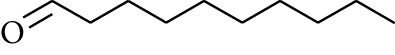	[23]
89	Aldoketone	Citronella	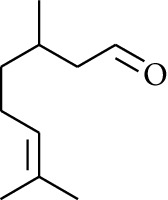	[23]
90	Aldoketone	2-Octenal	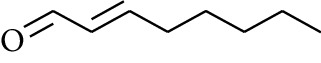	[21]
91	Aldoketone	Octanal	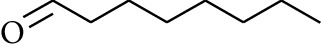	[26]
92	Aldoketone	Acetone	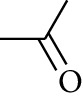	[26]
93	Acid	L*-*Bornyl acetate	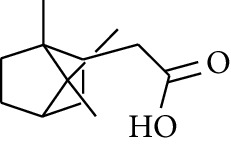	[15]
94	Acid	Geranic acid	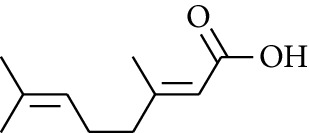	[15]
95	Acid	Undecanoic acid	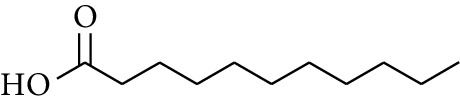	[16]
96	Ester	Neryl acetate	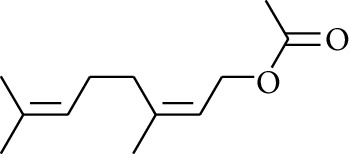	[15]
97	Ester	Methyl 11-(cyclopent-2-enyl)undecanoate	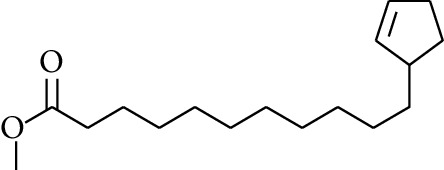	[15]
98	Ester	Geranyl propionate	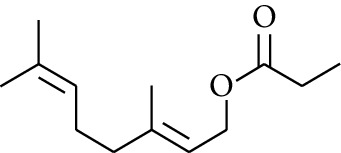	[15]
99	Ester	endo-Bornyl acetate	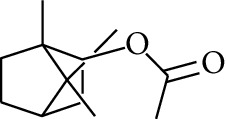	[15]
100	Ester	sec-Butyl acetate	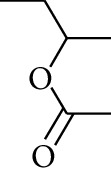	[15]
101	Ester	3,7-Dimethyl-2,6-octadienyl acetate	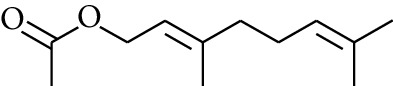	[15]
102	Ester	Neryl propionate	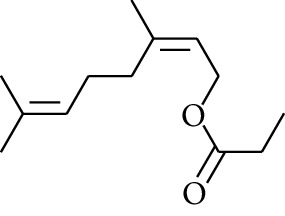	[15]
103	Ester	Geraniol formate	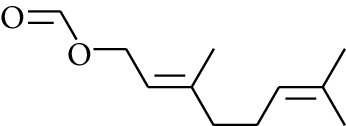	[15]
104	Ester	Myrtenyl acetate	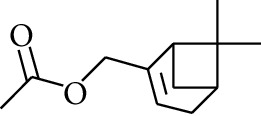	[15]
105	Ester	Geranyl acetate	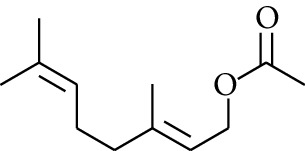	[15]
106	Ester	Formic acid ethyl ester	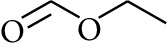	[16]
107	Ester	Ethyl butanoate	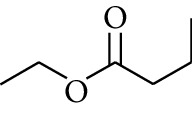	[17]
108	Ester	Citronellyl acetate	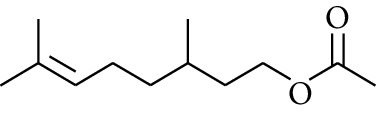	[17]
109	Ester	Heptyl acetate	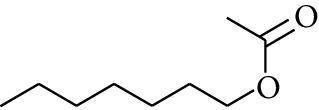	[17]
110	Ester	Methyl acetate	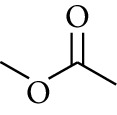	[18]
111	Ester	Ethyl acetate	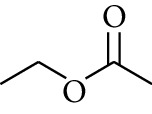	[18]
112	Ester	Butyl acetate	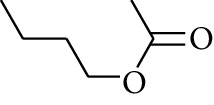	[21]
113	Ester	2-Octyl acetate	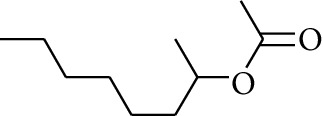	[21]
114	Fat hydrocarbon	allo-Aromadendrene	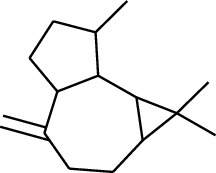	[15]
115	Fat hydrocarbon	*β*-Sesquiphellandrene	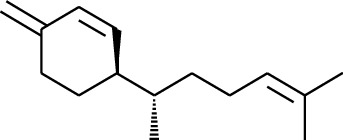	[15]
116	Fat hydrocarbon	*α*-Cedrene	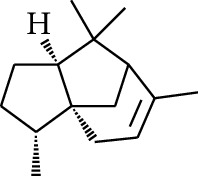	[15]
117	Fat hydrocarbon	*β*-Thujene	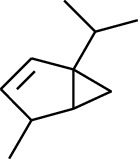	[15]
118	Fat hydrocarbon	Cadina-5,8-diene	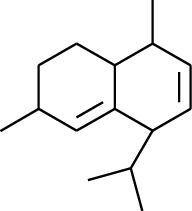	[15]
119	Fat hydrocarbon	Bicyclo[2.2.1]heptane	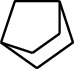	[15]
120	Fat hydrocarbon	*(E)*-2,7-Dimethyloct-3-en-5-yne	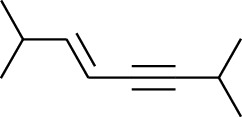	[15]
121	Fat hydrocarbon	*(Z)*-2,6-Dimethylocta-2,6-diene	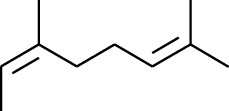	[15]
122	Fat hydrocarbon	*(E)*-3,7-Dimethylocta-1,3,6-triene	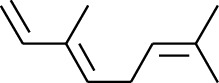	[15]
123	Fat hydrocarbon	*β*-Phellandrene	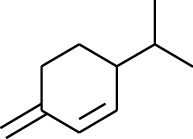	[15]
124	Fat hydrocarbon	*α*-Bergamotene	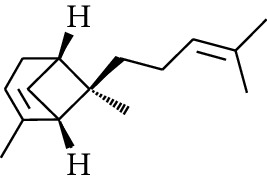	[15]
125	Fat hydrocarbon	*α*-Gurjunene	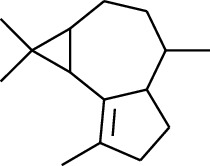	[15]
126	Fat hydrocarbon	Sabinene	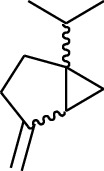	[15]
127	Fat hydrocarbon	(+)-Cyclosativene	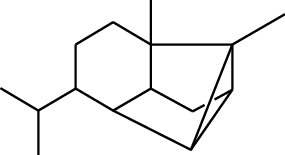	[15]
128	Fat hydrocarbon	*(Z)-β*-Farnesene	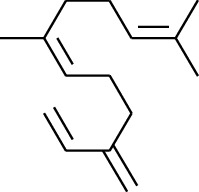	[15]
129	Fat hydrocarbon	*(E)-β*-Farnesene	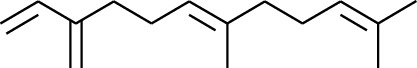	[15]
130	Fat hydrocarbon	(*Z*,*Z*)*-α*-Farnesene	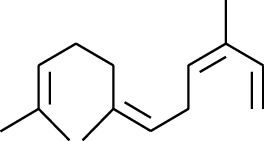	[15]
131	Fat hydrocarbon	Zingiberene	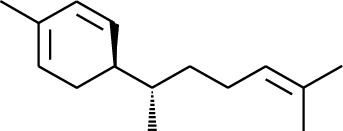	[15]
132	Fat hydrocarbon	*α*-Farnesene	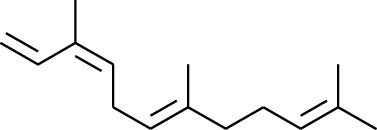	[15]
133	Fat hydrocarbon	(*E*)-5-Methylocta-1,6-diene	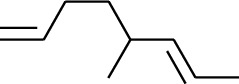	[15]
134	Fat hydrocarbon	5-Methyloct-3-yne	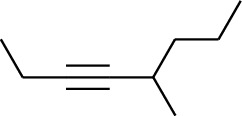	[15]
135	Fat hydrocarbon	7-Methylocta-3,4-diene	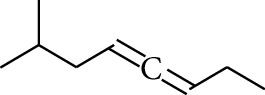	[15]
136	Fat hydrocarbon	*γ*-Elemene	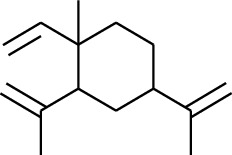	[15]
137	Fat hydrocarbon	*γ*-Humulene	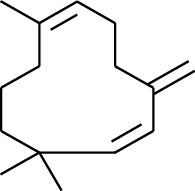	[15]
138	Fat hydrocarbon	Thujopsene	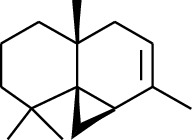	[15]
139	Fat hydrocarbon	*β*-Elemene	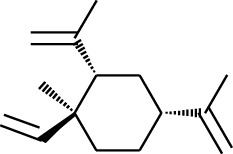	[15]
140	Fat hydrocarbon	*β*-Bisabolene	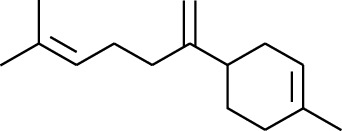	[15]
141	Fat hydrocarbon	*α*-Pinene	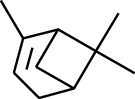	[15]
142	Fat hydrocarbon	*β*-Pinene	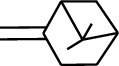	[15]
143	Fat hydrocarbon	Caryophyllene	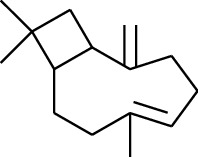	[15]
144	Fat hydrocarbon	*β*-Caryophyllene	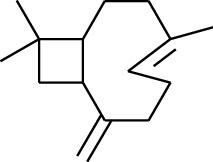	[15]
145	Fat hydrocarbon	Tricyclene	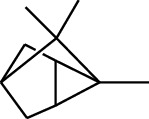	[15]
146	Fat hydrocarbon	Moslene	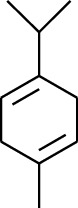	[15]
147	Fat hydrocarbon	Cedrene	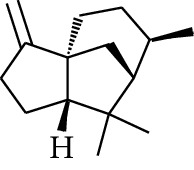	[15]
148	Fat hydrocarbon	(*–*)-allo-Aromadendrene	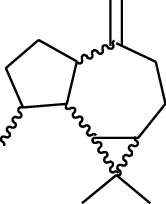	[15]
149	Fat hydrocarbon	Neoclovene	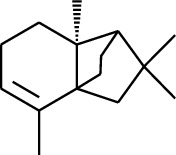	[15]
150	Fat hydrocarbon	3-Octyne	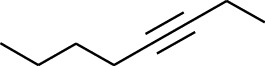	[15]
151	Fat hydrocarbon	1-Octene	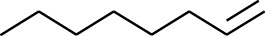	[15]
152	Fat hydrocarbon	*β*-Myrcene	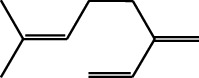	[15]
153	Fat hydrocarbon	*β*-Eudesmene	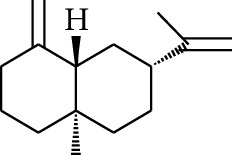	[15]
154	Fat hydrocarbon	Eudesma-3,7(11)-diene	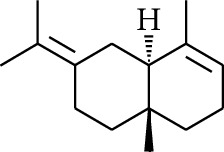	[15]
155	Fat hydrocarbon	Caryophyllene	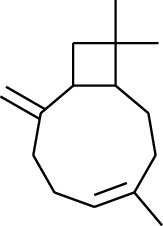	[15]
156	Fat hydrocarbon	Bicyclo[3.1.1]heptane	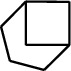	[15]
157	Fat hydrocarbon	1-Cyclopropylpentane	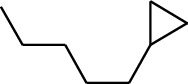	[15]
158	Fat hydrocarbon	3-Carene	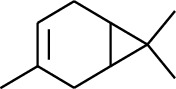	[15]
159	Fat hydrocarbon	2-Carene	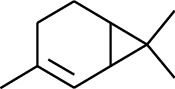	[15]
160	Fat hydrocarbon	(+)-Aromadendrene	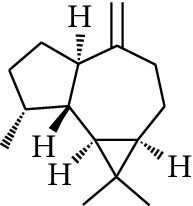	[15]
161	Fat hydrocarbon	Fenchene	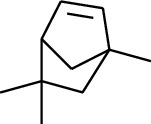	[16]
162	Fat hydrocarbon	*δ-*Elemene	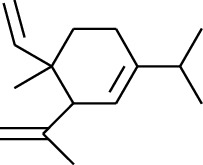	[17]
163	Fat hydrocarbon	D-Limonene	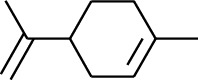	[18, 22]
164	Fat hydrocarbon	*β*-Phellandrene	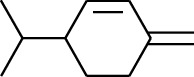	[18, 26]
165	Fat hydrocarbon	10-Epizonarene	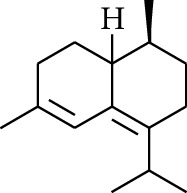	[18]
166	Fat hydrocarbon	Octane	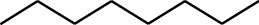	[18]
167	Fat hydrocarbon	Nonane	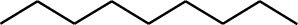	[18]
168	Fat hydrocarbon	*α*-Bergamotene	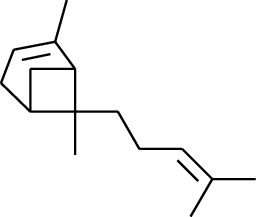	[19]
169	Fat hydrocarbon	*β*-Bisabolene	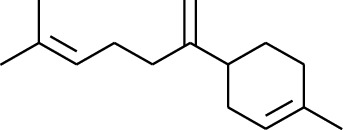	[20]
170	Fat hydrocarbon	*τ*-Epi-*α*-selinene	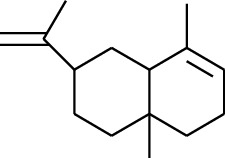	[20]
171	Fat hydrocarbon	4-Carene	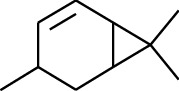	[22]
172	Fat hydrocarbon	Camphene	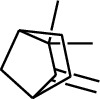	[23]
173	Fat hydrocarbon	*α*-Phellandrene	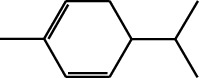	[23]
174	Fat hydrocarbon	*(Z)*-3,7-Dimethylocta-1,3,6-triene	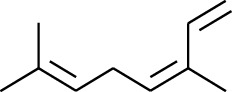	[27]
175	Fat hydrocarbon	Germacrene	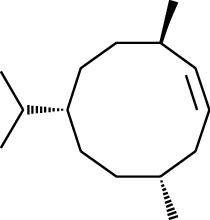	[27]
176	Fat hydrocarbon	*δ*-Cadinene	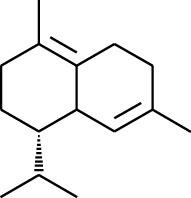	[26]
177	Fat hydrocarbon	*α*-Cubebene	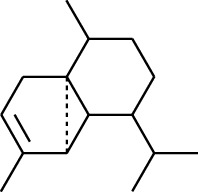	[26]
178	Fat hydrocarbon	*α*-Copaene	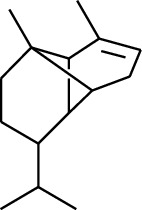	[26]
179	Arene	*α*-Curcumene	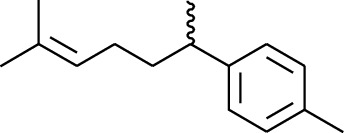	[15]
180	Arene	2-Isopropyltoluene	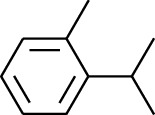	[15]
181	Arene	*o*-Cymene	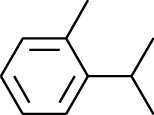	[15]
182	Arene	Styrene	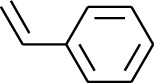	[17]
183	Arene	Methylbenzene	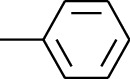	[17]
184	Arene	Cumene	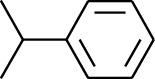	[18]
185	Arene	*p-*Cymene	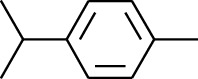	[19]
186	Others	*p*-Cymen-8-ol	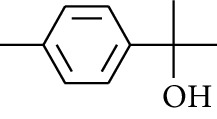	[15]
187	Others	2-Acetoxy-1,8-cineole	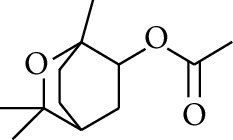	[17]
188	Others	Diethyl sulphide	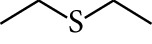	[18]
189	Others	Ethyl isopropyl sulphide	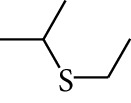	[18]
190	Others	Methyl allyl sulphide	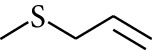	[18]
191	Others	Dibutyl phthalate	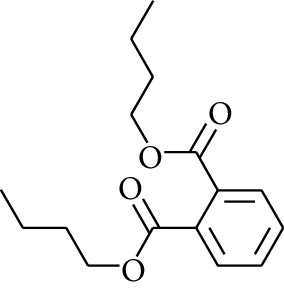	[20]
192	Others	2-(3′-Methyl-2′-butenyl)-3-methylfuran	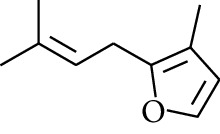	[21]
193	Others	Isoeugenol	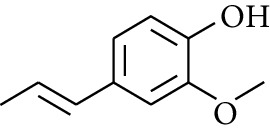	[21]
194	Others	2-(2′,3′-Epoxy-3′-methylbutyl)-3-methylfuran	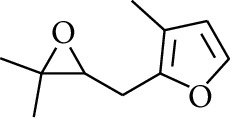	[21]

**Table 2:** Gingerols in ZOR.

No.	Name	Structure	R	R_1_	R_2_	*n*	Reference
195	3-Gingerol	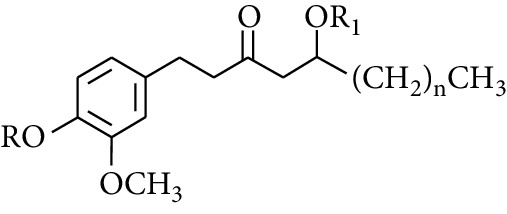	H	H	—	1	[30]
196	4-Gingerol		H	H	—	2	[30]
197	5-Gingerol		H	H	—	3	[30]
198	6-Gingerol		H	H	—	4	[31, 32]
199	8-Gingerol		H	H	—	6	[31, 33]
200	10-Gingerol		H	H	—	8	[33]
201	12-Gingerol		H	H	—	10	[31, 32]
202	5-Methoxy-6-gingerol		H	CH_3_	—	4	[34]
203	Acetoxy-4-gingerol		H	COCH_3_	—	2	[34]
204	Acetoxy-6-gingerol		H	COCH_3_	—	4	[35]
205	Acetoxy-8-gingerol		H	COCH_3_	—	6	[34]
206	Acetoxy-10-gingerol		H	COCH_3_	—	8	[34]
207	4-Gingeryl methyl ether		CH_3_	H	—	2	[34]
208	6-Gingeryl methyl ether		CH_3_	H	—	4	[34]
209	6-Gingeryl methyl ether acetate		CH_3_	COCH_3_	—	4	[34]
210	6-Gingeryl diacetate		COCH_3_	COCH_3_	—	4	[36]
211	8-Gingeryl diacetate		COCH_3_	COCH_3_	—	6	[36]
212	10-Gingeryl diacetate		COCH_3_	COCH_3_	—	8	[36]
213	Zingerone	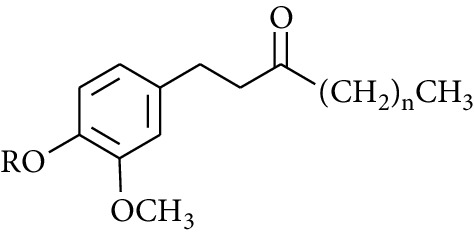	H	—	—	0	[36]
214	1-Paradol		H	—	—	1	[20]
215	2-Paradol		H	—	—	2	[20]
216	3-Paradol		H	—	—	3	[20]
217	4-Paradol		H	—	—	4	[20]
218	6-Paradol		H	—	—	6	[36, 37]
219	7-Paradol		H	—	—	7	[20]
220	8-Paradol		H	—	—	8	[36]
221	9-Paradol		H	—	—	9	[20]
222	10-Paradol		H	—	—	10	[36]
223	11-Paradol		H	—	—	11	[34]
224	13-Paradol		H	—	—	13	[34]
225	Methyl-6-paradol		CH_3_	—	—	6	[34, 38]
226	Methyl-8-paradol		CH_3_	—	—	8	[39]
227	Zingerone acetate		COCH_3_	—	—	0	[36]
228	6-Paradyl monoacetate		COCH_3_	—	—	6	[34]
229	8-Paradyl monoacetate		COCH_3_	—	—	8	[36]
230	6-Paradyl benzoate		COPh	—	—	6	[36]
231	1-Dehydro-3-gingerdione	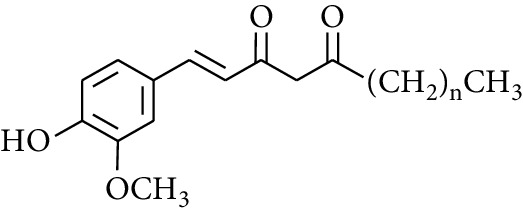	—	—	—	1	[34]
232	1-Dehydro-6-gingerdione		—	—	—	4	[35, 40]
233	1-Dehydro-8-gingerdione		—	—	—	6	[35, 41]
234	1-Dehydro-10-gingerdione		—	—	—	8	[37, 42]
235	12-Dehydrogingerdione		—	—	—	10	[43]
236	6-Gingerdione	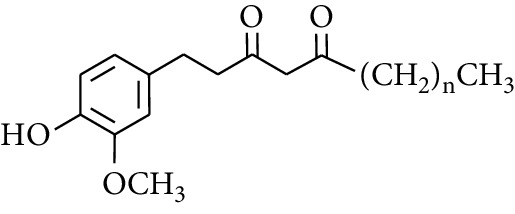	—	—	—	4	[44]
237	10-Gingerdione		—	—	—	8	[45]
238	4-Shogaol	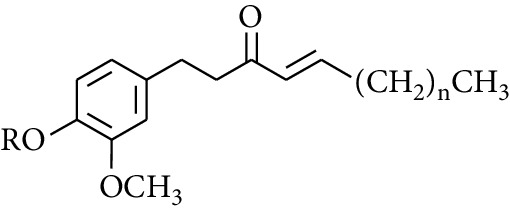	H	—	—	2	[30, 44]
239	5-Shogaol		H	—	—	3	[39]
240	6-Shogaol		H	—	—	4	[35, 37]
241	8-Shogaol		H	—	—	6	[36, 37]
242	10-Shogaol		H	—	—	8	[36, 37]
243	12-Shogaol		H	—	—	10	[30, 44]
244	Methyl-4-shogaol		CH_3_	—	—	2	[39]
245	Methyl-6-shogaol		CH_3_	—	—	4	[34]
246	Methyl-8-shogaol		CH_3_	—	—	6	[34]
247	4-Gingerdiol	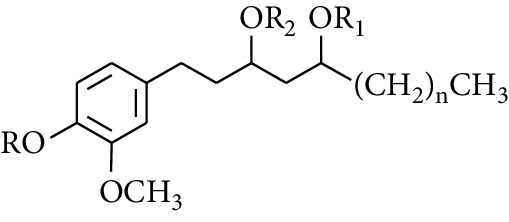	H	H	H	2	[30]
248	6-Gingerdiol		H	H	H	4	[44]
249	8-Gingerdiol		H	H	H	6	[44]
250	10-Gingerdiol		H	H	H	8	[44]
251	5-Acetoxy-4-gingerdiol		H	COCH_3_	H	2	[34]
252	5-Acetoxy-6-gingerdiol		H	COCH_3_	H	4	[46]
253	5-Acetoxy-7-gingerdiol		H	COCH_3_	H	5	[34]
254	Diacetoxy-4-gingerdiol		H	COCH_3_	COCH_3_	2	[46, 47]
255	Diacetoxy-6-gingerdiol		H	COCH_3_	COCH_3_	4	[46, 47]
256	Methyl-5-acetoxy-4-gingerdiol		CH_3_	COCH_3_	H	2	[34]
257	Methyl-5-acetoxy-6-gingerdiol		CH_3_	COCH_3_	H	4	[34]
258	Methyl diacetoxy-4-gingerdiol		CH_3_	COCH_3_	COCH_3_	2	[34]
259	Methyl diacetoxy-6-gingerdiol		CH_3_	COCH_3_	COCH_3_	4	[34]
260	Methyl diacetoxy-10-gingerdiol		CH_3_	COCH_3_	COCH_3_	8	[34]
261	6-Dihydroparadol	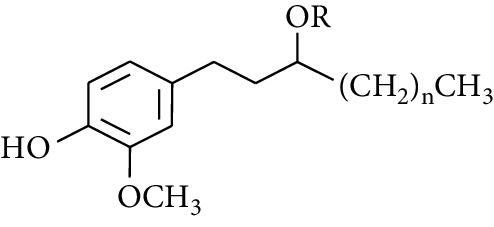	H	—	—	6	[34]
262	Acetoxy-6-dihydroparadol		Ac	—	—	6	[34]
263	1-(4′-Hydroxy-3′-methoxypheny-l)-7-octen-3-one	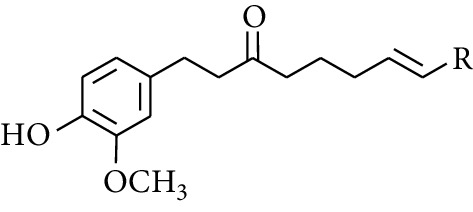	H	—	—	—	[34]
264	1-(4′-Hydroxy-3′-methoxypheny-l)-7-decen-3-one		CH_2_CH_3_	—	—	—	[34]
265	1-(4′-Hydroxy-3′-methoxypheny-l)-7-dodecen-3-one		(CH_2_)_3_CH_3_	—	—	—	[34]
266	4-Isogingerol	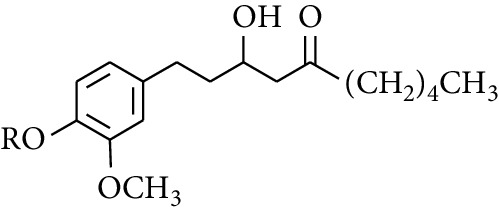	H	—	—	2	[34]
267	6-Isogingerol		H	—	—	4	[48]
268	Methyl-6-isogingerol		CH_3_	—	—	4	[34]
269	6-Zingerine	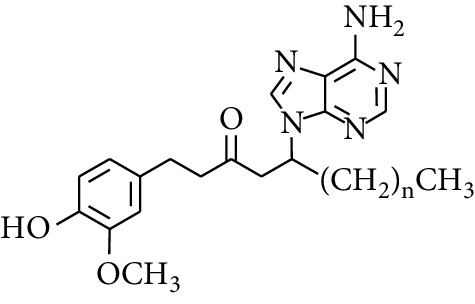	—	—	—	4	[49]
270	8-Zingerine		—	—	—	6	[49]
271	10-Zingerine		—	—	—	8	[49]
272	3-Dihydro-6-demethoxy shogaol	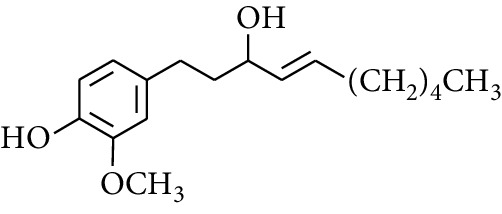	—	—	—	—	[34]
273	6-Isoshogaol	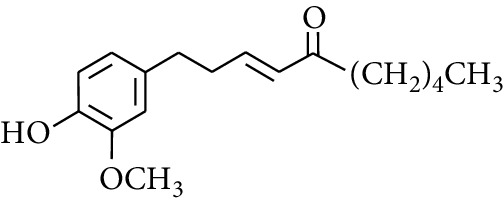	—	—	—	—	[44]
274	Dehydrozingerone	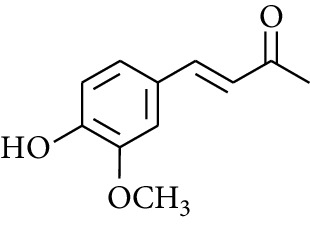	—	—	—	—	[36]
275	1-Dehydro-3-dihydro-10-gingerdione	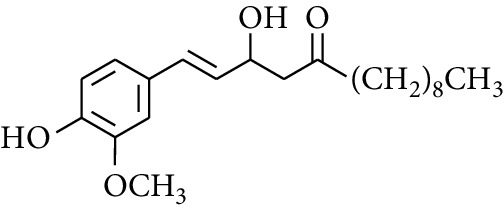	—	—	—	—	[34, 44]
276	(*Z*)-10-Isoshogaol	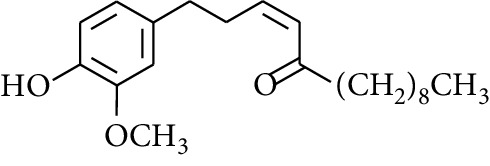	—	—	—	—	[48]
277	(*E*)-10-Isoshogaol	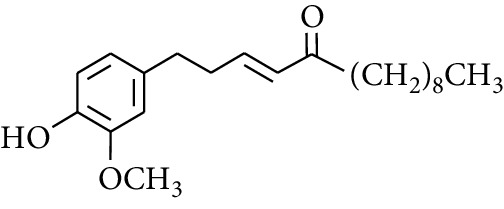	—	—	—	—	[48]
278	*β*-Sitosterol	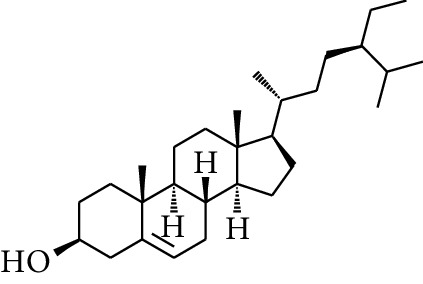	—	—	—	—	[50, 51]
279	Tetracosanoic acid	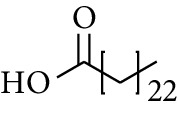	—	—	—	—	[50, 52]

**Table 3:** Diarylheptanoids in ZOR.

No.	Name	Structure	R_1_	R_2_	R_3_	Reference
280	5-Hydroxy-1-(4′-hydroxy-3′-methoxyphenyl)-7-(4″-hydroxyphenyl)heptan-3-one	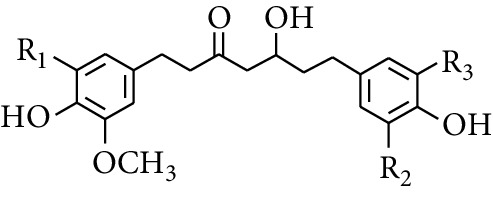	H	H	H	[32]
281	5-Hydroxy-1,7-bis(4′-hydroxy-3′-methoxyphenyl)heptan-3-one		H	OCH_3_	H	[32]
282	7-(3′,4′-Dihydroxy-5′-methoxyphenyl)-5-hydroxy-1-(4″-hydroxy-3″-methoxyphenyl)heptan-3-one		H	OCH_3_	OH	[54]
283	5-Hydroxy-7-(4′-hydroxy-3′,5′-dimethoxyphenyl)-1-(4″-hydroxy-3″-methoxyphenyl)heptan-3-one		H	OCH_3_	OCH_3_	[32]
284	5-Hydroxy-1-(4′-hydroxy-3′,5′-dimethoxyphenyl)-7-(4″-hydroxy-3″-methoxyphenyl)heptan-3-one		OCH_3_	OCH_3_	H	[55]
285	5-Hydroxy-1,7-bis(4′-hydroxy-3′,5′-dimethoxyphenyl)heptan-3-one		OCH_3_	OCH_3_	OCH_3_	[32]
286	*(E)*-7-(3′,4′-Dihydroxyphenyl)-1-(4″-hydroxy-3″-methoxyphenyl)hept-4-en-3-one	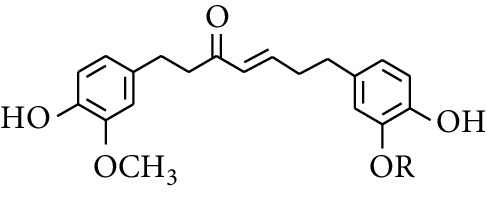	H	—	V	[55]
287	1,7-bis(4′-Hydroxy-3′-methoxyphenyl)-4-heptene-3-one		CH_3_	—	—	[55, 56]
288	3,5-Dihydroxy-1,7-bis(4′-hydroxy-3′-methoxyphenyl)heptane	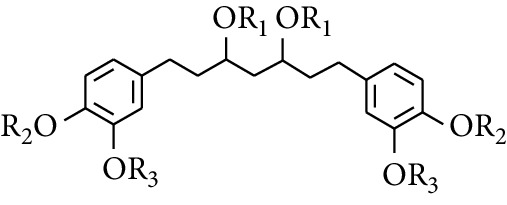	H	H	CH_3_	[54–56]
289	1,7-bis(3′,4′-Dihydroxyphenyl)-3,5-diacetate heptane		COCH_3_	H	H	[55]
290	1,7-bis(4′-Hydroxy-3′-methoxyphenyl)-3,5-diacetate heptane		COCH_3_	H	CH_3_	[55–57]
291	1,7-bis(4′-Methoxy-3′-acetatephenyl)-3,5-diacetate heptane		COCH_3_	CH_3_	COCH_3_	[55]
292	1,7-bis(3′,4′-Diacetatephenyl)-3,5-diacetate heptane		COCH_3_	COCH_3_	COCH_3_	[55]
293	5-(6-(4-Hydroxy-3-methoxyphenethyl)-4-hydroxy-tetrahydro-2H-pyran-2-yl)-3-methoxybenzene-1,2-diol	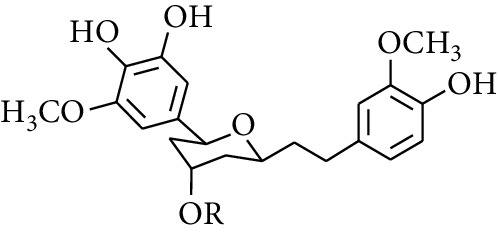	H	—	—	[54, 58]
294	2-(4′-Hydroxy-3′-methoxyphenethyl)-6-(3″,4″-dihydroxy-5″-methoxyphenyl)-tetrahydro-2H-pyran-4-yl acetate		COCH_3_	—	—	[58]
295	7-(3′,4′-Dihydroxyphenyl)-1-(4″-hydroxy-3″-methoxyphenyl)-3,5-diacetate heptane	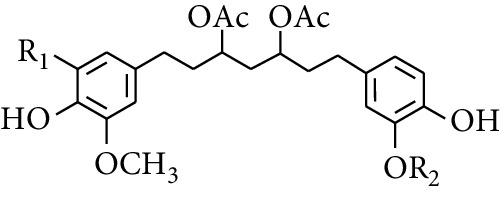	H	H	—	[55]
296	7-(4′-Hydroxy-3′-methoxyphenyl)-1-(4″,5″-dihydroxy-3″-methoxyphenyl)-3,5-diacetate heptane		OH	CH_3_	—	[54]
297	7-(4′-Hydroxy-3′-methoxyphenyl)-1-(4″-hydroxy-5″-methyl-3″-methoxyphenyl)-3,5-diacetate heptane		CH_3_	CH_3_	—	[55]
298	7-(4′-Hydroxy-3′-methoxyphenyl)-1-(4″-hydroxy-3″,5″-dimethoxyphenyl)-3,5-diacetate heptane		OCH_3_	CH_3_	—	[55, 56]
299	5-(6-(4-Hydroxyphenethyl)-4-hydroxy-tetrahydro-2H-pyran-2-yl)-3-methoxybenzene-1,2-diol	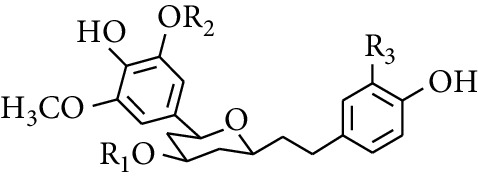	H	H	H	[25]
300	5-(6-(4-Hydroxy-3-methoxyphenethyl)-4-hydroxy-tetrahydro-2H-pyran-2-yl)-3-methoxybenzene-1,2-diol		H	H	OCH_3_	[54, 58]
301	5-(6-(4-Hydroxy-3-methoxyphenethyl)-4-hydroxy-tetrahydro-2H-pyran-2-yl)-2-hydroxy-3-methoxyphenyl acetate		H	COCH_3_	OCH_3_	[58]
302	2-(4′-Hydroxy-3′-methoxyphenethyl)-6-(3″,4″-dihydroxy-5″-methoxyphenyl)-tetrahydro-2*H*-pyran-4-yl acetate		COCH_3_	H	OCH_3_	[58]
303	1,7-bis(4′-Hydroxy-3′-methoxyphenyl)-5-oxoheptan-3-yl acetate	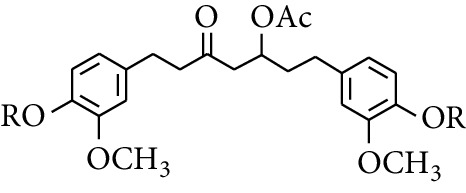	H	—	—	[57]
304	1,7-bis(3′-Methoxy-4′-acetatephenyl)-5-oxoheptan-3-yl acetate		COCH_3_	—	—	[57]
305	1,7-bis(4′-Hydroxy-3′-methoxyphenyl)-3,5-heptadione	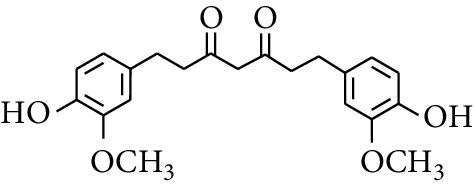	—	—	—	[34]
306	(*1E*,*6E*)-1,7-bis(4-Hydroxy-3-methoxyphenyl)hepta-1,6-diene-3,5-dione	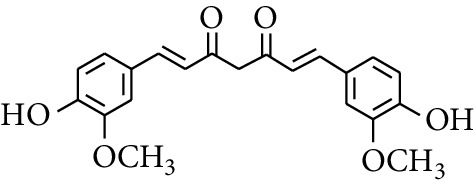	—	—	—	[56]
307	2,4-bis(3,4-Dihydroxyphenethyl)pentanedioic acid	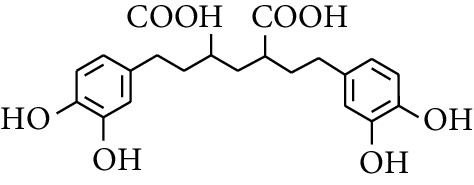	—	—	—	[59]
